# Structural brain differences in recovering and weight-recovered adult outpatient women with anorexia nervosa

**DOI:** 10.1186/s40337-021-00466-w

**Published:** 2021-09-03

**Authors:** Brooks B. Brodrick, Adrienne L. Adler-Neal, Jayme M. Palka, Virendra Mishra, Sina Aslan, Carrie J. McAdams

**Affiliations:** 1 Department of Psychiatry, University of Texas Southwestern Medical Center, 5323 Harry Hines Blvd., Suite BL6.110, Dallas, TX 75390-9070 USA; 2 Department of Internal Medicine, University of Texas Southwestern Medical Center, 5323 Harry Hines Blvd., Dallas, TX 75390-9070 USA; 3 Advance MRI LLC, Frisco, TX 75034 USA

**Keywords:** Eating disorders, Social cognition, Anorexia nervosa, Autism, Depression, Anxiety, Bulimia nervosa, Self-perception, Gray matter

## Abstract

**Background:**

Anorexia nervosa is a complex psychiatric illness that includes severe low body weight with cognitive distortions and altered eating behaviors. Brain structures, including cortical thicknesses in many regions, are reduced in underweight patients who are acutely ill with anorexia nervosa. However, few studies have examined adult outpatients in the process of recovering from anorexia nervosa. Evaluating neurobiological problems at different physiological stages of anorexia nervosa may facilitate our understanding of the recovery process.

**Methods:**

Magnetic resonance imaging (MRI) images from 37 partially weight-restored women with anorexia nervosa (pwAN), 32 women with a history of anorexia nervosa maintaining weight restoration (wrAN), and 41 healthy control women were analyzed using FreeSurfer. Group differences in brain structure, including cortical thickness, areas, and volumes, were compared using a series of factorial f-tests, including age as a covariate, and correcting for multiple comparisons with the False Discovery Rate method.

**Results:**

The pwAN and wrAN cohorts differed from each other in body mass index, eating disorder symptoms, and social problem solving orientations, but not depression or self-esteem. Relative to the HC cohort, eight cortical thicknesses were thinner for the pwAN cohort; these regions were predominately right-sided and in the cingulate and frontal lobe. One of these regions, the right pars orbitalis, was also thinner for the wrAN cohort. One region, the right parahippocampal gyrus, was thicker in the pwAN cohort. One volume, the right cerebellar white matter, was reduced in the pwAN cohort. There were no differences in global white matter, gray matter, or subcortical volumes across the cohorts.

**Conclusions:**

Many regional structural differences were observed in the pwAN cohort with minimal differences in the wrAN cohort. These data support a treatment focus on achieving and sustaining full weight restoration to mitigate possible neurobiological sequela of AN. In addition, the regions showing cortical thinning are similar to structural changes reported elsewhere for suicide attempts, anxiety disorders, and autistic spectrum disorder. Understanding how brain structure and function are related to clinical symptoms expressed during the course of recovering from AN is needed.

**Supplementary Information:**

The online version contains supplementary material available at 10.1186/s40337-021-00466-w.

## Plain English summary

Anorexia nervosa is a life-threatening mental illness defined in part by an inability to maintain a body weight in the normal range. Malnutrition and low weight are factors typically present in the anorexia nervosa and can affect brain structure. We conducted a detailed analysis of brain structure using Freesurfer, focusing on regional cortical thicknesses, areas, and volumes, in adult outpatient women with anorexia nervosa. The study included both a partially weight-restored cohort with anorexia nervosa, a cohort sustaining a healthy body weight with history of anorexia nervosa, and a healthy comparison cohort. Reduced cortical thicknesses were observed in eight regions, primarily in the frontal lobe and cingulate for the cohort recently with anorexia nervosa but only one frontal region in the weight-maintained cohort. These data emphasize the importance of sustained weight-restoration for adult women with anorexia nervosa. Further, the impacted neural regions have been associated with impulsivity, attention, self-regulation, and social interactions in other clinical cohorts, suggesting that these neuropsychological processes may warrant study in patients recovering from anorexia nervosa. Future work should consider whether these factors have clinical relevance in the outpatient treatment of adults with anorexia nervosa.

**Supplementary Information:**

The online version contains supplementary material available at 10.1186/s40337-021-00466-w.

## Introduction

Anorexia nervosa (AN) is a life-threatening mental illness characterized by low body weight and impaired sense of self-worth, with an over emphasis placed that one’s body shape and size determines one’s value as a person. Recovery from AN often takes years and 5–10% of individuals with AN will die [1] due to its severe medical complications or suicide [2]. Even after intensive treatment and restoration of normal body weight, relapse is common in adults with AN [3]. Avoidance of weight loss immediately following treatment was associated with long-term weight maintenance [4]; whereas psychological measures at discharge were not predictive of clinical course [5]. A critical gap in AN research has been difficulty identifying psychological and cognitive factors ‬that enable ‬successful ‬weight ‬maintenance ‬after ‬acute weight ‬restoration. As such, improving our understanding of neurobiological changes that occur during this critical time period could contribute to our understanding of the course of illness, and potentially elucidate treatment targets important during recovery from AN. ‬‬‬‬‬‬‬‬‬‬‬‬‬‬‬‬‬‬‬‬‬‬‬‬‬‬‬‬‬‬‬‬‬‬‬‬‬‬‬‬‬‬‬‬‬‬‬‬‬‬‬‬‬‬‬‬‬‬‬‬‬‬‬‬‬‬‬‬‬‬‬‬‬‬‬‬‬‬‬‬‬‬‬‬‬‬‬‬‬‬‬‬‬‬‬‬‬‬‬‬‬‬

Structural brain analyses can measure gray and white matter volumes, cortical thicknesses and areas, as well as subcortical volumes for regions and structures within the brain. In one meta-analysis of global brain structure in AN, Seitz and colleagues [6] characterized changes in gray matter volumes, white matter volumes, and cerebrospinal fluid volume while considering acutely ill AN, short-term weight-recovered AN, and long-term recovered AN. All measures were reduced in the acutely ill group, reductions in gray matter and cerebrospinal fluid were observed in the short-term weight recovered cohort, and no significant differences were observed in the long-term weight restored cohort. In considering regional differences in brain structure, many studies have compared underweight AN cohorts early or even before beginning treatment to a healthy comparison group [7–12], generally reporting reductions in cortical thickness in some regions in AN. Some studies have considered both underweight AN as well as weight-restored individuals, with most differences resolved after weight-restoration [13–16], with one machine-learning study suggesting increases in orbitofrontal and insula as well as decreases in superior frontal regions might be a state biomarker of AN [17]. Considered as a whole, the literature strongly supports the hypothesis that structural brain differences in AN are closely tied to weight.

Importantly, as AN is defined by alterations in eating behaviors and changes in body mass index (BMI), weight and the disorder are always confounded [18]. Because acute malnutrition alters brain volumes [19], examination of partially-weight restored individuals with AN may reduce the impact of acute malnutrition and provide a better understanding of neurobiological processes relevant during the early stages of recovering. Clinically, the greatest risk of weight loss and resumption of eating disorder behaviors occurs in the first year after intensive treatment [3], supporting a need to better understand individuals at this stage of illness. Regional brain differences have only been analyzed for a single cohort of young, partially-weight restored participants with AN responding well to recent intensive treatment. This cohort showed normalization of cortical thinning [14] and improvement in most local gyrification indices [20] in relation to a healthy comparison cohort. These two studies shared participants, and deployed a longitudinal design such that only those individuals achieving marked weight-restoration in a short time period were included. Thus, there remains a gap in our knowledge, as there are no studies that have evaluated regional brain structure for adult outpatients in the process of recovering from AN.

There is a clinical need to develop a better understanding of neuropsychological factors during partial weight restoration as many patients spend substantial time in this state. In a recent 5-year annual follow-up study of adult patients with AN following intensive weight rehabilitation, only 46% maintained a BMI greater than 18.5 throughout follow-up, with only 17% sustaining both a BMI greater than 18.5 and normalization of EDE-Q scores [5]. These data are consistent with that from several naturalistic studies that have followed outpatients with AN, reporting remission rates from 39 to 42% [21–23]. One large cohort study of adults with AN has found that 31% were recovered after 9 years and 62% recovered at 22 years [24]. In a clinical trial reporting on time-to-relapse after acute weight-restoration for adults with AN, less than 30% maintained a BMI over 18.5 for twelve months [25], with relapse most common in the first two months [26]. In concert, these data suggest that maintaining weight-restoration and achieving recovery is a slow and unpredictable process for adults with AN. Developing a better neurobiological understanding at this stage of the illness may help in designing and targeting treatments to improve outcomes.

Given this need to identify neuropsychological factor that may impact illness course, we compared cortical thicknesses, areas, and volumes in 37 partially weight-restored women with anorexia nervosa (pwAN), 32 adult women with a history of AN and maintaining weight-restoration (wrAN), and 41 healthy comparison (HC) adult women. The goal of this study is to increase our understanding of neuropsychological problems present during outpatient treatment of adult women with AN. We anticipated that clinical symptoms (BMI, depression, eating, self-esteem) would be worse in the pwAN relative to the wrAN and HC cohorts. Based on the extant eating disorder literature in structural brain analyses, we hypothesized that frontal and cingulate regions would show reduced cortical thickness in the pwAN cohort relative to the HC cohort, and expected normalization for all cortical regions in the wrAN cohort.

## Methods

### Participants

The participants for this study were drawn from two separate IRB-approved neuroimaging protocols that included different functional brain imaging tasks [27–31]; this is the first publication examining structural brain data collected from these protocols. For this study, all participants were required to be female, and between age 18 and 46 years. The HC cohort was required to have no current DSM-IV psychiatric illnesses, with a BMI between 19 and 30. Substance use disorders, bipolar disorder, and psychotic disorders were exclusionary for all cohorts; participants with major depressive disorder and anxiety disorders were permitted. Current use of mood stabilizers and antipsychotic medications were exclusionary but antidepressants were permitted.

For the AN cohorts, participants with AN from the earlier studies were classified into two cohorts based on both BMI at the time of the scan, as well as the duration at which the person had sustained a BMI greater than 18.5. This study defined partially weight restored AN (pwAN) as individuals whose body mass index (BMI) had been less than 18.5 in prior six months, but were at a stable or increasing weight over the last month, and whose BMI at the time of the scan remained under 19.5. Most of the pwAN cohort were recruited from and scanned soon after completion of intensive treatment programs, including inpatient, residential, partial hospital, and intensive outpatient; all individuals whose BMI was under 17.5 were scanned within a month of discharge, and continued to show a stable or increasing BMI over that period. To qualify for the weight-restored cohort (wrAN), participants had a lifetime-diagnosis of AN, a BMI greater than 19.5, and had not had a BMI under 18.5 at any time in at least six months.

### Clinical measures

Both protocols used the Structured Clinical Interview for DSM-IV (SCID) psychiatric disorders to establish eating disorder diagnosis and comorbidities. The timeline from the SCID provided an age of onset for both AN cohorts and age of recovery for the wrAN cohort. The clinical assessments common to both protocols included Eating Attitudes Test-26 (EAT) and its three subscales dieting (EAT-D), bulimia and food preoccupation (EAT-B) and oral control (EAT-O) [32], and the Quick Inventory of Depressive Symptoms [33]. Self-esteem was assessed with the Self-Liking and Self-Competence (SLSC) Questionnaire [34] and social problem-solving with the short version of the Social Problem Solving Inventory-R Short (SPSI-R [35], which provides five subscales that include positive problem orientation (PPO), negative problem orientation (NPO), rational problem solving (RPS), impulsivity/carelessness style (ICS), and avoidance style (AS).

### MRI methods

Structural brain scans were acquired using a 3 T Philips Achieva MRI scanner. A high-resolution T1-weighted image called Magnetization Prepared Rapid Acquisition of Gradient-Echo (MPRAGE) sequence was collected with the following parameters: FOV = 256 × 256 × 160 mm^3^, TR/TE = 8.1 ms/3.7 ms, flip angle = 12, 160 sagittal slices, voxel size = 1 × 1 × 1 mm^3^ and duration of 4 min. The cortical surface was reconstructed for each subject using the FreeSurfer 6.0.1 pipeline [36, 37]. Briefly, each T1-weighted image was spatially and intensity normalized to Talairach Atlas. Volumetric segmentation and subcortical labelling were then performed on the normalized images. The gray matter (GM) and white matter (WM) boundaries were then automatically identified and reconstructed into a mesh of over 150,000 tessellated vertices for surface measures. Gyral anatomy was then aligned to a standard spherical template using surface convexity and curvature measures.

An estimate of contrast to noise ratio (CNR) in white matter was computed for every subject using the FreeSurfer’s QA tools (https://surfer.nmr.mgh.harvard.edu/fswiki/QATools). Only data with a CNR > 15 were utilized for further analysis. Furthermore, the datasets that passed the CNR threshold but had a poorly reconstructed surface, we performed manual correction following the guidelines of the FreeSurfer developers. Thickness (mm) and area (mm^2^) measures were extracted for all cortical regions identified in Desikan-Killiany atlas [38] along with default volume (mm^3^) measures for all cortical and subcortical regions were extracted for all participants, using the FreeSurfer tools, aparcstats2table and asegstats2table, respectively.

### Statistical analyses

Prior to conducting statistical analyses, data were examined for the presence of outliers and subjected to assumption testing. For demographic (i.e., race, ethnicity, age, years of education and clinical outcomes (i.e., BMI, QIDS total score, EAT-D score, EAT-B score, EAT-O score, and EAT total score), group differences were examined using chi-squared analyses (for categorical variables) or a one-way ANOVA (for continuous variables). For models that did not meet homogeneity of variance assumptions (i.e., QIDS, EAT-D, EAT-B, EAT-O, and EAT total), Welch’s robust ANOVA was used. Tukey post-hoc tests were conducted on statistically significant results. These analyses were conducted using IBM SPSS Statistics version 25.0 (Armonk, NY: IBM Corp.).

To determine group differences in thickness, area, and volumes, a series of factorial *F*-tests were completed. All models included a group factor (i.e., HC, pwAN, wrAN), with HC as the reference group, while age was entered as a covariate. Results were considered statistically significant at *p* < 0.05. To correct for multiple comparisons among the 166 factorial *F*-tests, the Benjamini and Hochberg (1995) false discovery rate (FDR) method was applied [39]. An FDR Q-value of 0.15 was chosen in order to remain conservative yet still detect true positives. Six families of comparisons were identified based upon the type of structural data (i.e. thickness, area, and volume) as well as hemisphere (left, right); FDR corrections were conducted within each family separately. Models with omnibus *p*-values that passed FDR correction with a statistically significant group effect at *p* < 0.05 were retained. Finally, Cohen’s *d* values were calculated for models that remained statistically significant after applying FDR corrections. The FDR analyses were conducted in R [40] using the lm and p.adjust functions.

Statistically significant models obtained with the analytic sample (*n* = 110) used in the primary analyses, were analyzed a second time using a new analytic sample (*n* = 103) with those having a BMI < 17 removed. This was to ensure that the findings derived from the primary analyses were not driven by the seven individuals in the pwAN cohort with a BMI < 17 at the time of the scan.

## Results

### Demographic and clinical measures

Participants in the study from the first study protocol included 12 pwAN, 8 wrAN, and 13 healthy comparison (HC) women, and from the second protocol included 25 pwAN, 24 wrAN, and 28 HC women. The full sample includes 37 pwAN, 32 wrAN, and 41 HC women. Antidepressants were permitted, with 16 in the pwAN cohort, 11 in the wrAN, and 1 in the HC cohort. For comorbidities, 13 from the pwAN and 15 from the wrAN had recurrent major depressive disorder; and, eight each from the pwAN and wrAN cohort had a comorbid anxiety disorder (generalized anxiety disorder, panic disorder, or agoraphobia). None of the HC sample met criteria for a eating disorders (lifetime), current anxiety disorders, or had a current episode of major depressive disorder; one HC had a history of recurrent major depressive disorder. Participants were largely non-Hispanic (n = 101) and Caucasian (n = 91), although there were a few Native American (n = 2), Asian (n = 13) and Black (n = 4) participants; no differences in race (*X*^2^(4, 110) = 1.189, *p* = 0.888) or ethnicity (*X*^2^(2, 110) = 0.102, p > 0.99) were observed in the comparisons across the three cohorts. Age and education did not differ across the three groups (Table 1).

**Table 1:** Demographic and clinical measures by cohort

		Clinical cohort						Statistical comparisons		
		pwAN (n = 37)		wrAN (n = 32)		HC (n = 41)				
		Mean	SD	Mean	SD	Mean	SD	*F*	*df* (b/w)	*p*
Age (years)		25.4	6.75	29.4	8.27	26.7	6.20	2.908	2/107	.059
Education (years)		15.08	2.23	15.44	2.36	15.76	2.29	0.846	2/107	.432
Body mass index		**17.83**^**a**^	1.17	22.09	2.33	23.17	3.12	82.138	2/59.827	** < .001**
Quick inventory of depression		**6.92**^**a**^	4.68	**6.53**^**a**^	5.15	2.22	2.20	22.026	2/56.078	** < .001**
Eating attitudes test (EAT) total		**31.41**^**a**^	19.80	**23.78**^**a**^	14.69	3.78	3.93	59.504	2/48.294	** < .001**
EAT	Dieting subscale	**16.89**^**a**^	11.81	**14.56**^**a**^	9.07	2.29	3.13	49.189	2/50.754	** < .001**
EAT	Bulimia and food subscale	**7.22**^**ab**^	5.32	**4.81**^**ab**^	3.14	0.41	0.81	56.898	2/47.564	** < .001**
EAT	Oral control subcale	**6.81**^**ab**^	5.16	**4.16**^**ab**^	4.52	1.12	1.44	25.875	2/50.407	** < .001**
Self-esteem (SLSC)										
SLSC	Self-liking	**17.89**^**a**^	6.68	**17.44**^**a**^	6.49	31.41	6.55	54.54	2/107	** < 0.001**
SLSC	Self-competence	**22.97**^**a**^	6.29	**23.47 **^**a**^	3.93	28.90	4.98	15.10	2/107	** < 0.001**
Social problem solving inventory-revised (SPSI-R)		**11.54**^**a**^	2.84	**12.27**^**a**^	2.43	14.66	2.58	13.91	2/104	** < 0.001**
SPSI-R	Positive problem orientation	**9.30 **^**ab**^	3.62	**11.28 **^**ab**^	3.55	13.24	2.43	13.62	2/104	** < 0.001**
SPSI-R	Negative problem orientation	**11.54**^**ab**^	4.39	**8.81 **^**ab**^	3.57	4.66	3.62	28.95	2/104	** < 0.001**
SPSI-R	Rational problem solving	10.41	3.45	10.41	3.62	12	3.49	2.45	2/104	.091
SPSI-R	Impulsivity/carelessness style	3.03	3.15	5.03	3.88	3.66	3.13	3.03	2/104	.053
SPSI-R	Avoidance style	7.43^a^	4.76	6.44^a^	4.39	3.63	4.45	6.84	2/104	**.002**

Bold values indicate statistically significant Tukey post hoc comparisons at *p* < .05 with ^a^different from HC and ^b^differences between pwAN and wrAN cohorts

Clinically, results from the one-way ANOVA (Table 1) revealed statistically significant group differences in BMI with the pwAN cohort lower than the other two groups (HC vs. pwAN: *p* < 0.001, *d* = 2.266; wrAN vs. pwAN: *p* < 0.001, *d* = 2.311). There were no differences in the age of onset of anorexia nervosa across the pwAN and wrAN cohorts, with both groups developing the illness in late adolescence (Age of Onset, years, pwAN 17.0 [3.57], wrAN 16.4 [5.92], F(1,67) = 0.290, *p* = 0.592). A similar proportion of both cohorts had the restricting and binge-purge subtypes of AN (pwAN, 21 restrict, 16 binge-purge; wrAN 19 restrict, 13 binge-purge; *X*^2^(1,69) = 0.048, *p* = 0.826). The age of recovery for the wrAN cohort averaged 26.4 [7.1] years. Ten individuals in the pwAN cohort and two individuals in the wrAN cohort had BMIs between 18.5 and 19.5; the individuals in pwAN cohort who reported maintaining a BMI over 18.5 had done so for 2.6 [1.35] months while the two in the wrAN cohort had BMIs over 18.5 for an average of 7.5 [0.5] months.

Many differences in the self-report measures of depression, eating, self-esteem, and social problem solving were observed across the three groups (Table 1). Statistically significant group differences were present in the EAT, with the pwAN group having the highest scores on the EAT and all its subscales: EAT total score (HC vs. pwAN: *p* < 0.001, *d* =  − 1.936; HC vs. wrAN: *p* < 0.001, *d* =  − 1.861). Of note, the pwAN and wrAN also differed from each other on the bulimia subscale of the EAT (EAT-B; pwAN vs. wrAN: *p* = 0.016, *d* = 0.552), and the oral control subscale of the EAT (EAT-O; pwAN vs. wrAN: *p* = 0.017, *d* = 0.547). Both the pwAN and wrAN cohorts had elevated depression scores relative to the HC cohort, and did not differ from each other (QIDS; HC vs. pwAN: *p* < 0.001, *d* =  − 1.285; HC vs. wrAN: *p* < 0.001, *d* =  − 1.089). Similarly, both the pwAN and wrAN cohorts had lower self-esteem measures than the HC cohort, including self-liking (SL; HC vs. pwAN: *p* < 0.001, *d* = 13.32; HC vs. wrAN: *p* < 0.001, *d* = 13.98) and self-competence (SC; HC vs. pwAN: *p* < 0.001, *d* = 5.93; HC vs. wrAN: *p* < 0.001, *d* = 5.43), but did not differ from each other. Although the total SPSI-R scores were lower for both pwAN and wrAN relative to HC (SPSI-R; HC vs. pwAN: *p* < 0.001, *d* = 3.11; HC vs. wrAN: *p* = 0.001, *d* = 2.39) they did not differ for each other. However, on both the positive problem orientation (PPO) and the negative problem orientation (NPO) subscales of the SPSI-R, all three groups differed from each other (PPO: HC vs. pwAN: *p* < 0.001, *d* = 3.94; HC vs. wrAN: *p* < 0.038, *d* = 1.96; wrAN vs. pwAN *p* < 0.036, *d* = 1.984; NPO: HC vs. pwAN: *p* < 0.001, *d* =  − 6.88; HC vs. wrAN: *p* < 0.001, *d* =  − 4.16; wrAN vs. pwAN *p* < 0.014, *d* =  − 2.73). No other differences in the SPSI-R were significant across cohorts.

### Brain structure

No statistically significant differences were observed in cerebral white matter volume (CWM, pwAN 290,777 mm^3^, wrAN 299,594 mm^3^, HC 300,015 mm^3^; *F*[3, 104] = 1.796; *p* = 0.153), total gray matter volume (TGM, pwAN 442,391 mm^3^, wrAN 452,821 mm^3^, HC 458,506 mm^3^; *F*[3, 104] = 0.882; *p* = 0.453) or subcortical gray matter volume (SGM pwAN 39,351 mm^3^, wrAN 40,233 mm^3^, HC 40,517 mm^3^; *F*[3, 104] = 0.534; *p* = 0.660).

**Fig. 1:**
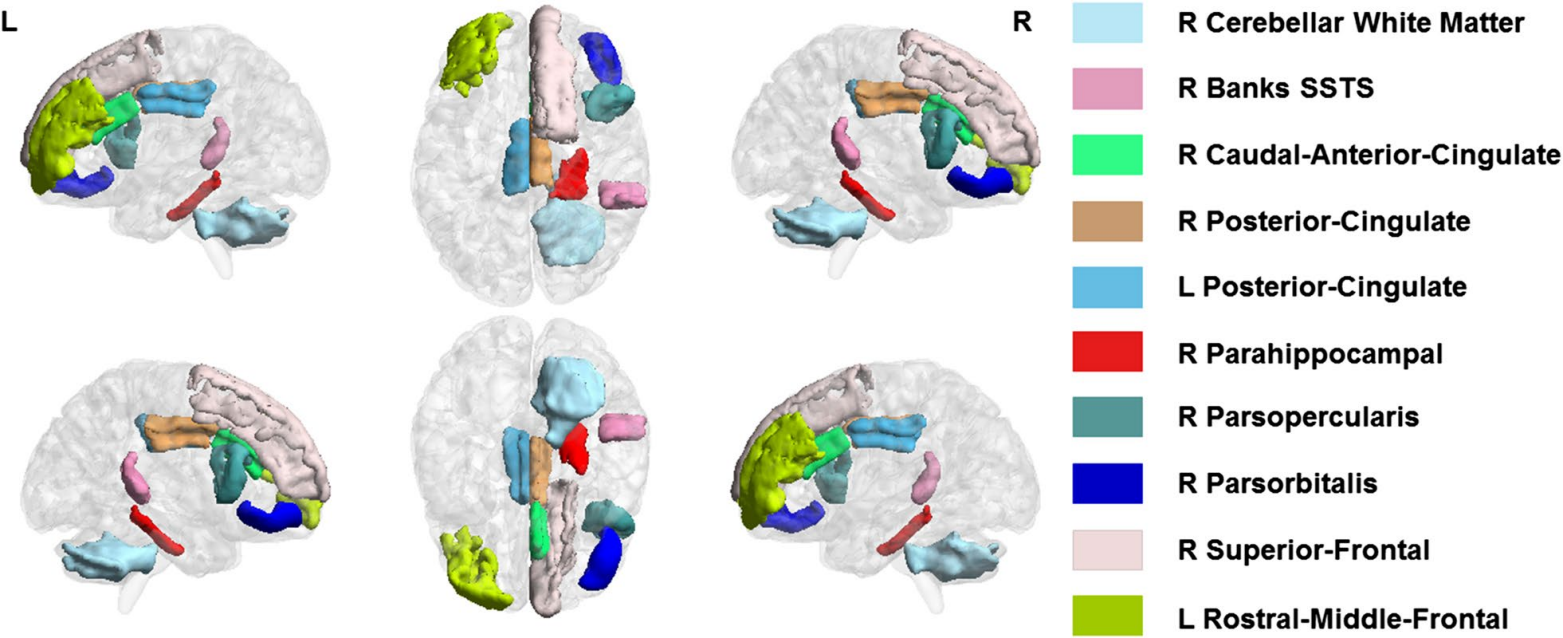
Nine cortical thicknesses and one volume showed significant differences across the three cohorts. Each region is colored slightly differently. Of note, the R parahippocampal gyrus (red) was thicker in the pwAN cohort relative to the HC cohort; the R pars orbitalis (blue) was thinner in both the pwAN and wrAN relative to the HC cohort; the posterior cingulate thicknesses were thinner in the pwAN cohort relative to both the wrAN and HC cohorts; the remaining cortical thicknesses were thinner for the pwAN cohort relative to the HC cohort

Group differences survived FDR corrections for nine cortical thicknesses and one volume difference (Fig. 1; Table 2). These regions included: right bank SSTS (pwAN: β =  − 0.075, *p* = 0.021), right caudal anterior cingulate (pwAN: β =  − 0.131, *p* < 0.001), right parahippocampal (pwAN: β = 0.109, *p* = 0.036), right pars opercularis (pwAN: β =  − 0.057, *p* = 0.035), right pars orbitalis (wrAN: β =  − 0.081, *p* = 0.036; pwAN: β =  − 0.079, *p* = 0.034), right posterior cingulate (pwAN: β =  − 0.082, *p* = 0.002), left posterior cingulate (pwAN: β =  − 0.092, *p* = 0.006), left rostral middle frontal (pwAN: β =  − 0.059, *p* = 0.022), right superior frontal (pwAN: β =  − 0.077, *p* = 0.002), and right cerebellar white matter (pwAN: β =  − 848.929, *p* = 0.034). The effect sizes associated with these differences were medium to large, with the smallest difference in the right pars opercularis (Cohen’s d = 0.400) and the largest difference in the right caudal anterior cingulate (Cohen’s d = 0.799).

**Table 2:** Cortical thickness differences in cohorts

	Clinical group						Statistical comparisons				
	pwAN (n = 37)		wrAN (n = 32)		HC (n = 41)				Pairwise effect sizes		
	Mean	SD	Mean	SD	Mean	SD	*F*	*p*	Comparison		*Cohen’s d*
R Bank SSTS	**2.65**^**a**^	0.15	2.69	0.15	2.72	0.12	3.192	.027	pwAN	wrAN	− 0.267
										HC	** − 0.515**
									wrAN	HC	− 0.221
R Caudal Anterior Cingulate	**2.50 **^**a**^	0.13	2.57	0.18	2.63	0.19	5.979	< .001	pwAN	wrAN	− 0.446
										HC	** − 0.799**
									wrAN	HC	− 0.324
R Parahippocampal	**2.82 **^**a**^	0.22	2.76	0.22	2.70	0.25	4.987	.003	pwAN	wrAN	0.273
										HC	**0.510**
									wrAN	HC	0.255
R Parsopercularis	**2.77 **^**a**^	0.13	2.78	0.12	2.82	0.12	5.763	.001	pwAN	wrAN	− 0.080
										HC	** − 0.400**
									wrAN	HC	− 0.333
R Parsorbitalis	**2.83 **^**a**^	0.18	**2.80 **^**a**^	0.17	2.90	0.15	5.398	.002	pwAN	wrAN	0.171
										HC	** − 0.423**
									wrAN	HC	** − 0.624**
R Posterior Cingulate	**2.49 **^**ab**^	0.10	2.57	0.12	2.57	0.12	5.324	.002	pwAN	wAN	** − 0.724**
										HC	** − 0.724**
									wrAN	HC	0.000
L Posterior Cingulate	**2.48 **^**ab**^	0.16	2.57	0.13	2.57	0.14	3.374	.021	pwAN	wrAN	** − 0.617**
										HC	** − 0.599**
									wrAN	HC	0.000
L Rostral Middle Frontal	**2.53 **^**a**^	0.12	2.55	0.12	2.58	0.10	3.866	.011	pwAN	wrAN	− 0.167
										HC	** − 0.453**
									wrAN	HC	− 0.272
R Superior Frontal	**2.83 **^**a**^	0.11	2.86	0.10	2.90	0.10	7.935	< .001	pwAN	wrAN	− 0.285
										HC	** − 0.666**
									wrAN	HC	− 0.400

Bold values indicate statistically significant comparisons at *p* < .05, with ^a^different from HC and ^b^differences between pwAN and wrAN cohorts. For all *F*-tests, *df*(b/w) = 3/104

The majority of the cortical thickness differences were in the right hemisphere (7/9), and in the frontal lobe [5]. One region, the right pars orbitalis, was thinner in both the pwAN and wrAN cohorts. Seven regions were thinner in the pwAN cohort than the HC, with two of these regions, the left and right posterior cingulate, also thinner in the pwAN cohort compared to the wrAN cohort. One region, the right parahippocampal gyrus, was thicker in the pwAN cohort than the HC cohort. The right cerebellar white matter was smaller in the pwAN than the other two cohorts (Right Cerebellum White Matter mm^3^, pwAN 9139(1417) wAN 10,239(1986) HC 10,045(1788), *F[3,104]* = 4.060, *p* = 0.009; pwAN vs. HC, *d* =  − 0.562; pwAN vs. wrAN, *d* =  − 0.638; wrAN vs. HC, *d* = 0.102).

To confirm that results from the primary analyses were not driven by the *n* = 7 individuals with a BMI < 17, *f*-tests were repeated for the above mentioned statistically significant models, after removing *n* = 7 from the analytic sample (Additional file 1: Table S1). The group effect in six models remained statistically significant with an analytic *n* = 103: right bank SSTS (pwAN: β =  − 0.081, *p* = 0.020), right caudal anterior cingulate (pwAN: β =  − 0.146, *p* < 0.001), right parahippocampal (pwAN: β = 0.109, *p* = 0.037), right pars orbitalis (wrAN: β =  − 0.081, *p* = 0.042; pwAN: β =  − 0.091, *p* = 0.024), right posterior cingulate (pwAN: β =  − 0.084, *p* = 0.003), and right superior frontal (pwAN: β =  − 0.066, *p* = 0.010).

## Discussion

Clinical, psychological and structural differences in the brain were compared across three groups: women partially weight-restored and appropriate for outpatient treatment of AN (pwAN), women with history of AN but currently weight-restored (wrAN), and women without EDs (HC). The pwAN and wrAN cohorts both had significantly more depression, eating symptoms, and lower self-esteem scores than the HC cohort; in addition, the pwAN cohort had higher levels of disordered eating, as well as more negative (NPO on SPSI-R) and less positive (PPO on SPSI-R) orientations in social problem solving than the wrAN cohort. Several structural neural differences were observed in group comparisons, including eight neural regions with reduced cortical thickness in the pwAN cohort relative to the HC cohort, with one of these regions, the right pars orbitalis, also showing reduced cortical thickness in the wrAN cohort. No differences in cortical surface areas were observed, and only one volume measure, the right cerebellar white matter volume, was reduced in the pwAN cohort compared to the other groups. The effect sizes for all observed cortical differences were medium to large.

The differences in cortical thinning observed in the pwAN cohort are more pronounced than those observed in the only other study that has evaluated a cohort of partially weight restored AN. That cohort was younger (average age 15.5 years), assessed on average three months after beginning an intensive weight restoration program from a low starting weight, and restricted to participants able to gain substantial weight during the treatment [14]. Nevertheless, differences in local gyrification index were observed after weight-restoration for this younger cohort, and those differences were in right-sided frontal and temporal regions [20]; the same areas we report to be thinner in our pwAN cohort. Considering that our sample was about 10 years older, these data support a hypothesis that frontal and temporal regions may be slow to recover following an acute episode of AN.

The first step in recovery from AN is weight-restoration and requires sufficient caloric intake to maintain an individual at a healthy body weight. Most previous studies examining weight-recovered cohorts with AN suggest structural brain differences resolve after sustained weight restoration [13–16]. Unfortunately, clinical studies have shown that achieving and maintaining weight restoration is quite difficult for adult women with AN [5, 21, 24–26]. Clinical differences are seldom reported for comparisons of both pharmacological and psychological outpatient treatments for adults with AN [41, 42], suggesting a critical need to better understand treatments that are effective at this stage of AN. In this discussion, we consider the differences observed in the pwAN and wrAN cohorts, in relation to both common roles for these regions in healthy individuals, and prior studies of these areas in eating disorder and other psychiatric illnesses that can be comorbid with AN. These comparisons may be helpful in guiding future research to improve our understanding of the neuropsychological changes involved in the recovery process for adult outpatients with AN.

Only one cortical thickness, the right pars orbitalis, differed in both pwAN and wrAN. In studies of healthy human behaviors, this area has been associated with impulse-control [43], emotional communication [44, 45], and social exclusion [46]. Numerous studies have observed functional brain differences in the inferior frontal gyrus and insula in both AN and BN, a region anatomically close and overlapping with this area [47, 48]. Previously, from a subset of this sample, we reported that reduced activations of the right inferior frontal gyrus were observed in an AN cohort relative to a HC cohort when participants were engaged in evaluation of social interactions relative to conducting a non-social, physical evaluation about interactions [27]. In adolescents with bulimia nervosa, reduction in cortical thickness of the right inferior frontal gyrus was previously hypothesized to be a potential trait marker of bulimia nervosa, as more pronounced reduction correlated with increased frequency of purging episodes [49]. The observed cortical thinning in this region for both the pwAN and wrAN cohorts lend additional credence to consideration of this area as a potential trait marker important in eating disorders.

Here, we also observed social cognitive differences between the pwAN and wrAN cohorts in two subscales of the social problem solving inventory, with more negative and less positive problem-solving orientations, as assessed with the NPO and PPO subscales of the SPSI-R, for the pwAN cohort relative to wrAN, and both cohorts relative to HC. Clinically, problems with mentalization and non-verbal social communication have been observed in patients with AN [50–52], and interventions that target social-emotional function are being explored in anorexia nervosa. Cognitive remediation and emotional skill training (CREST) augments treatment of severe AN, addressing social anhedonia, alexithymia, and quality of life [53, 54]. Improvements in eating disorder symptoms, anxiety, and depression were observed with a brief group therapy intervention targeting self-attributions and perspective-taking for outpatients with eating disorders [55]. Since these social cognitive processes (PPO and NPO) differ both in relation to illness (both pwAN and wrAN differ from HC), and the degree of difference is impacted by illness state (pwAN is worse than wrAN), these data support a hypothesis that social cognition is altered during the course of recovering from AN.

Many of the structural differences observed in the pwAN cohort, including reduced cortical thickness in the right banks of the superior temporal gyrus, right pars opercularis, right pars orbitalis, right superior frontal gyrus, bilateral posterior cingulate, and diminished right cerebellar volume, are in areas frequently linked to social cognitive behaviors and emotional reasoning. The right pars opercularis is closely tied to reasoning and social cognition [56]. Both the inferior frontal gyrus (includes pars opercularis and orbitalis) and the superior temporal sulcus are engaged during tasks that involve empathy, imitation, and theory of mind in healthy participants [57]. When self-relevant information is attributed to another person, both the superior frontal gyrus and posterior cingulate are activated [58]. In previously published functional MRI studies from subsets of participants from this sample, altered activations during social self-evaluations were reported for the posterior cingulate, precuneus and dorsal anterior cingulate [28, 30], while diminished responses were observed in the temporoparietal junctions in AN during both a social attribution task [27] and a social neuroeconomic game [29].

Cerebellar volume deficits have been amongst the most consistent regional volume differences present in AN [6]. Reduced cerebellar volume has been associated with duration of illness in AN [59]. Reduced right cerebellum volume has also been associated with autistic behaviors in both animal models and humans with autistic spectrum disorders, with the right cerebellar crus showing strong functionally connectivity with both the superior frontal gyrus and posterior cingulate in neurotypical adults [60]. Recently, the cerebellum has been hypothesized to be essential for the adaptations and learning required for social behaviors [61].

Three regions in the cingulate were also thinner in pwAN including the right caudal anterior cingulate as well as the right and left posterior cingulate. Reduced cortical thickness in the cingulate has been one of the most common regional changes reported in AN [6]. The anterior cingulate is involved in value-based decision-making including social motivation and reward-based behaviors [62]. The posterior cingulate is a key node in the default mode network, and associated with arousal, activities that require internally-directed attention and consideration of oneself in relation to others [63–65]. Problems in self-regulation, both regarding recognition of internal emotional as well as physical states, are well-established in AN [66]. Also potentially relevant to AN, thinning of the cingulate cortex has been associated with low vitamin D in normal aging [67]. Of note, both posterior cingulate cortical thicknesses were the only regions for which the pwAN cohort differed from both the wrAN and HC cohorts, further supporting a hypothesis that the cingulate may be particularly vulnerable to malnutrition.

One of only two differences on the left side of the brain was a thinner left rostral middle frontal region, an area associated with emotional stress [68]. Cortical thickness in this area also mediates the relationship between expressed neuroticism and polygenic risk score for the trait of neuroticism [69]. This area has previously been reported as thinner in panic disorder [70] as well as in suicide attempts [71]. In the Sarkinaite’s examination of suicidality, cortical thicknesses were compared for both single attempt and multiple attempt patients, finding three regions that were thinner than the healthy comparison cohort in single attempt individuals and seventeen regions that were thinner in the multiple attempt individuals [71]. Several regions from both suicidality cohorts overlap with regions observed to be thinner in the pwAN cohort here. Suicidality is common in anorexia nervosa, with most deaths from the illness due to suicide [2].

Finally, one area, the right parahippocampal gyrus, was larger in the pwAN cohort relative to the other two cohorts. The parahippocampal gyrus has been associated with memory formation, exercise, and emotional regulation The exercise data may be most relevant to this sample, as the parahippocampal gyrus shows increased cortical thickness in relation to increased exercise in many different types of studies in both human and animal models (for review [72]). One study assessed hippocampal volume in women with AN in relation to excessive exercise, observing a larger hippocampal volume in women with AN engaged in excessive exercise that normalized after weight restoration [73]. Another potentially relevant study examined individuals with anxiety disorders undergoing cognitive behavioral therapy, finding reduced activations in the right parahippocampal gyrus were associated with improved clinical symptoms after treatment, with those authors proposing that this area may be involved in maintaining negative emotional states [74]. Both overexercise [75] and maintaining negative emotional states [76] are also common in AN.

There are many limitations to this study. First, this study combined structural data obtained from two different functional imaging studies to increase sample size, and relatively few clinical measures overlapped across the studies or differed in the pwAN and wrAN cohorts, preventing examination of whether structural changes could be directly related to clinical assessments. The clinical course of AN in adult patients is complex, and some factors that may be related to nutrition and the brain structure including duration at low body weight were not available. The study is moderately-sized, larger than most structural studies in the literature for AN, but not as large as many studies of other psychiatric illnesses. The limited sample size prevents consideration of the impact of antidepressant use or comorbid diagnoses on the results. Neither cohort included severely underweight individuals, so questions about the brain structure present in more severely malnourished individuals cannot be answered. This is a cross-sectional study of only adult women, so results should not be generalized to younger ages that are also common in AN, and its cross-sectional nature prevents assigning function and symptoms changes to the brain differences.

## Conclusions

These structural brain imaging data from outpatients recovering and recovered from AN provide new information about brain structure during the later stages of weight restoration and weight maintenance. Most importantly, nearly all of the structural changes were resolved in the wrAN cohort, supporting the importance of keeping a focus in treatment of AN on both achieving and maintaining a BMI greater than 19.5, approximately the third percentile for adult women in the United States. Even after excluding the lowest weight individuals (BMI < 17, 19% of the pwAN cohort), many structural differences remained, suggesting the neurobiological impact of AN resolves slowly. Areas showing cortical thinning in the pwAN cohort share similarities with structural changes previously reported in suicide attempts, anxiety disorders, and autistic spectrum disorder; more research evaluating these types of symptoms over time in patients with AN in concert with structural and functional neural data is needed. A broader set of neural, clinical and psychological symptom data may be helpful to establish how neurophysiological changes are related to clinical problems experienced by individuals recovering from AN.

## Supplementary Information

**Additional file 1.** Cortical Thickness Differences Excluding Low BMI Participants.

## Data Availability

Data and materials are available on request.

## Abbreviations

AN: Anorexia nervosa
pwAN: Partially-weight restored cohort with anorexia nervosa
wrAN: A 6-month minimum weight-restored cohort with lifetime history of AN
HC: The healthy comparison cohort
IRB: Institutional review board
