# Supplementary material for: Structural brain differences in recovering and weight-recovered adult outpatient women with anorexia nervosa
Source: J Eat Disord. 2021 Sep 3;9:108. doi: 10.1186/s40337-021-00466-w (PMC8414694; doi:10.1186/s40337-021-00466-w)
Supplement: Supplementary file 1 — Additional file 1. Cortical Thickness Differences Excluding Low BMI Participants. [file 40337_2021_466_MOESM1_ESM.docx]

Supplemental Materials For:

Structural brain differences in recovering and weight-recovered adult outpatient women with anorexia nervosa.

Table S1: Cortical Thickness Differences Excluding Low BMI

| Table S1. *Cortical Thickness Differences in Regions after Exclusion of Very Low BMI (< 17) from pwAN Cohort* | | | | | | | | |
| --- | --- | --- | --- | --- | --- | --- | --- | --- |
|  | **pwAN (n = 30)** | | **wrAN**  **(n = 32)** | | **HC**  **(n = 41)** | | **Statistical Comparisons** | |
|  | **Mean** | **SD** | **Mean** | **SD** | **Mean** | **SD** | ***F*** | ***p*** |
| R Bank SSTS | **2.66^a^** | 0.16 | 2.69 | 0.14 | 2.72 | 0.12 | 3.473 | .019 |
| R Caudal Anterior Cingulate | **2.50^a^** | 0.14 | 2.57 | 0.11 | 2.63 | 0.12 | 6.361 | .001 |
| R Parahippocampal | **2.84^a^** | 0.20 | 2.76 | 0.22 | 2.71 | 0.24 | 6.682 | <.001 |
| R Parsopercularis | 2.77 | 0.13 | 2.78 | 0.11 | 2.81 | 0.12 | 5.859 | .001 |
| R Parsorbitalis | **2.83^a^** | 0.19 | **2.80^a^** | 0.16 | 2.91 | 0.14 | 5.142 | .002 |
| R Posterior Cingulate | **2.49^ab^** | 0.10 | 2.57 | 0.12 | 2.56 | 0.12 | 5.139 | .002 |
| L Posterior Cingulate | 2.48 | 0.16 | 2.57 | 0.13 | 2.58 | 0.13 | 2.511 | .063 |
| L Rostral Middle Frontal | 2.54 | 0.12 | 2.55 | 0.12 | 2.59 | 0.10 | 4.138 | .045 |
| R Superior Frontal | **2.85^a^** | 0.11 | 2.86 | 0.10 | 2.90 | 0.11 | 6.057 | <.001 |
| *Note.* Bold values indicate statistically significant Tukey post hoc comparisons at p < .05 with ^a^ different from HC and ^b^ differences between pwAN and wrAN cohorts. For all *F*-tests, *df*(b/w) = 3/98. | | | | | | | | |
